# Toward the automated detection of behavioral changes associated with the post-weaning transition in pigs

**DOI:** 10.3389/fvets.2022.1087570

**Published:** 2023-01-04

**Authors:** Ilias Kyriazakis, Ali Alameer, Katarína Bučková, Ramon Muns

**Affiliations:** ^1^School of Biological Sciences, Institute for Global Food Security, Queen's University Belfast, Belfast, United Kingdom; ^2^Sustainable Agri-Food Science Division, Livestock Production Science Branch, Agri-Food and Biosciences Institute, Hillsborough, United Kingdom

**Keywords:** behavior, drinking, early warning, feeding, machine learning, pigs, post-weaning diarrhea, swine

## Abstract

We modified an automated method capable of quantifying behaviors which we then applied to the changes associated with the post-weaning transition in pigs. The method is data-driven and depends solely on video-captured image data without relying on sensors or additional pig markings. It was applied to video images generated from an experiment during which post-weaned piglets were subjected to treatments either containing or not containing in-feed antimicrobials (ZnO or antibiotics). These treatments were expected to affect piglet performance and health in the short-term by minimizing the risk from post-weaning enteric disorders, such as diarrhea. The method quantified total group feeding and drinking behaviors as well as posture (i.e., standing and non-standing) during the first week post-weaning, when the risk of post-weaning diarrhea is at its highest, by learning from the variations within each behavior using data manually annotated by a behavioral scientist. Automatically quantified changes in behavior were consistent with the effects of the absence of antimicrobials on pig performance and health, and manifested as reduced feed efficiency and looser feces. In these piglets both drinking and standing behaviors were increased during the first 6 days post-weaning. The correlation between fecal consistency and drinking behavior 6 days post weaning was relatively high, suggesting that these behaviors may have a diagnostic value. The presence or absence of in-feed antimicrobials had no effect on feeding behavior, which, however, increased over time. The approach developed here is capable of automatically monitoring several different behaviors of a group of pigs at the same time, and potentially this may be where its value as a diagnostic tool may lie.

## 1. Introduction

Piglet weaning, which occurs abruptly in the early stages of their life, is a critical stage in pig production. Weaning is associated with profound changes in the structure and function of the gastrointestinal tract, arising mainly from the abrupt transition from liquid milk to solid creep feed (1). Furthermore, weaning introduces several stress factors, including the move to a new farm environment that may affect the intestinal microflora and immune function of pigs negatively (2, 3). Such disturbances increase the risk of enteric disorders, particularly post-weaning colibacillosis (PWC), which is caused primarily by the proliferation of enterotoxigenic Escherichia coli (ETEC) (4). Such disorders are associated with post-weaning diarrhea (PWD).

Traditionally, the risk of enteric disorders in newly weaned pigs has been mitigated through the use of in-feed antimicrobial growth promoters (AGP), including the inclusion of prophylactic levels of zinc oxide (ZnO). However, concerns about the use of AGP in livestock systems have led to legislation that aims to reduce their use (5), including the banning of prophylactic ZnO in-feed inclusion from 2022. These concerns arise from AGP association with the potential for development of pathogen antimicrobial resistance and concerns about environmental impacts, amongst other things (6). Although the field of developing alternatives to AGP is a very active one, the expectation is that the risk of enteric disorders amongst piglets will increase during the post-weaning transition. Therefore, early detection of enteric disorders amongst weaned piglets will become even more important, as it will allow rapid action to enable treatment effectiveness and prevent further spread within a group (7). In our paper, we develop the theme of early detection of enteric disorders of weaned piglets through monitoring of behavior, based on the principle that behavioral changes that happen during a health challenge may have a diagnostic value (8). Due to the enormity of scale of pig production systems, this theme will be of value only if it is based on automated detection. The aim of this paper was to apply and evaluate an automated method, based on visual imaging, to monitor the behaviors of pigs that may have diagnostic value for the detection of enteric disorders of weaned pigs (9).

The automated method originates from the work of Alameer et al. (10, 11), but is developed further here to account for the challenge at hand. The method depends solely on image data without relying on sensors or additional pig marking and is trained to identify pig behaviors under the constantly challenging farm environment. It is data-driven, as it correctly learns the variations within each behavior of relevance, using data manually annotated by a behavioral scientist. We apply this method to quantify the behavior of piglets in the period immediately after weaning, in the presence and absence of in-feed AGP, which are expected to affect piglet performance in the short and longer term. We focus on the changes in four behaviors potentially associated with the post-weaning transition of pigs: feeding, drinking, standing and lying, and investigate their diagnostic value. Here we detail our hypotheses about how these piglet behaviors are expected to change during this phase. One of the most obvious consequences of post weaning enteric disorders associated with PWD, is a reduction in the feed intake of piglets (12, 13). Piglets start to consume small amounts of solid feed after weaning, but feed intake reduces once the condition develops. In some cases, there is complete cessation of feeding, frequently associated with other clinical signs. The reduction in feed intake would be expected to be associated with a reduction in water intake, but it is possible that piglets actually increase their water intake to compensate for the water loss during the diarrhea that accompanies post-weaning enteric disorders (14). Currently it is unclear whether changes in piglet drinking behavior have diagnostic value for the detection of post-weaning enteric disorders. Finally, most health challenges are associated with changes in animal standing, lying and posture (8): sick animals may spend more time lying and less time standing. In the case of the post-weaning enteric disorders this may be a direct consequence of the reduction in feed intake and the accompanying fever (1). The latter may be associated with huddling to keep warm.

## 2. Materials and methods

### 2.1. Study animals and management

The study was carried out at Agri-Food and Biosciences Institute's (AFBI) experimental farm, Hillsborough, Northern Ireland between August and November 2020. A total of 360 crossbred Duroc x (Large White x Landrace) piglets were monitored across six production batches (60 pigs/batch). Between birth and weaning at 28 days of age, piglets were housed in standard farrowing pens (2.3 x 1.5 m) with forward creep areas. From day 10 of age pigs were offered creep feed (17.9 MJ/kg digestible energy (DE), 21.55% crude protein (CP), 2.21% crude fiber (CF), 1.52% Lysine; Devenish Nutrition Ltd., Belfast, UK); the creep feed did not contain any antimicrobials.

All pigs were tail docked, with approximately 50% of the tail removed within 24 h of birth; male pigs were not castrated. The pigs were weaned and moved to nursery accommodation at 28 ± 1 days of age and remained there until day 69 ± 1 (10 weeks of age). Weaning piglet body weight (BW) was 9.47 kg ± 1.20 (Mean ± SD). In the nursery accommodation, pigs were housed in mixed-sex groups of 10 on plastic slatted pens (2.7 x 1.4 m), which were distributed amongst 2 rooms in a balanced manner. Groups were balanced according to BW, sex and as much as possible, sow parity. Each pen was provided with environmental enrichment in the form of a suspended wooden block and flavored plastic biting toy (Porcichew, Nutrapet Ltd., U.K.). Pen temperature was initially set at 28°C, but decreased 0.5°C/day, stabilizing at 21°C. Each room was mechanically ventilated and subject to natural lighting; artificial lighting was provided daily when routine farm procedures were carried out.

### 2.2. Dietary treatments

At weaning, pigs were offered one of three experimental treatments (T) consisting of a Starter 1 (S1) diet 89 (16.25 MJ/kg DE, 20% CP; 1.65% Lys, and 2.11% CF) for 13 days, followed by Starter 2 (S2) diet for 16 days 90 (16.25 MJ/kg DE, 20% CP, 1.54% Lys, and 2.29% CF). Treatments were:

- T1, diets S1 and S2 with added ZnO (2,500 mg/kg, PIGZIN, DSM Nutritional Products Ltd., UK);
- T2, diets S1 and S2 without any additional ZnO, considered the negative control;
- T3, diets S1 and S2 with added antibiotic (Apramycin, 100 mg/kg), which was considered as the positive control. Apramycin has been widely used in pig systems to control for post-weaning diarrhea of bacterial origin.

At the end of the starter treatment period, all pigs were offered the same grower diet (15.0 MJ/kg DE, 17.5% CP, 1.20% Lys, and 3.0% CF) up to the end of trial (10 weeks of age). Each pen was provided with a different treatment (2 pens/treatment/batch). Feed was offered *ad libitum* in a dry multi-space feeder (Etra Feeders, Northern Ireland, UK). Pigs also had *ad libitum* access to water provided through a nipple inside of a bowl (Echberg Drik-O-Mat drinker, Denmark).

### 2.3. Measurements taken

#### 2.3.1. Pig performance

BW was recorded for each animal at weaning (day 0), at the beginning and end of offering the S2 diet (days 13 and 29 post-weaning) and at the end of the trial (day 40 post-weaning). Total pen feed intake was also recorded at 13- and 40-days post-weaning. BW data were also used to calculate average daily gain (kg/d, ADG) for the time intervals: weaning 0–13 days and 0–40 days. The average feed intake per pig was calculated by dividing the pen level feed intake by the number of pigs in the pen. From this, we determined the average daily feed intake (kg/d, ADFI) per pig for the same periods of interest described for ADG. The feed conversion ratio (FCR) was calculated as the total amount of feed eaten per pen for the 13-and 40-day period divided by the respective pen ADG.

#### 2.3.2. Fecal consistency and dry matter content

Fecal consistency scoring was conducted at the pen level on days 5 and 12 post-weaning. Each pen was searched for fresh feces twice a day and each time given a fecal score according to its consistency and appearance using the fecal consistency score system ranging from 1 to 4 (1 = firm and shaped feces, 4 = liquid feces) as described by Marquardt et al. (15).

Fresh fecal samples were obtained from the floor of each pen on the same days for dry matter determination. Fecal samples were stored at 4°C until ready for processing. Dry matter (%) was determined by oven drying (Gallenkamp, UK) at 100°C for 24 h (16). We focused on measurements for 119 days 5 and 12 because PWD is usually observed during the first 2 weeks post weaning (1).

### 2.4. Manual and automated behavior observation

Video cameras (4 M.P. Fixed Bullet Network Cameras, HiLook IPC-B140H(-M), Hikvision, Hangzhou, China) were connected to a network video recorder (16 PoE 4K NVR; model: DS-7716NI-I4/16P) and installed onto the ceiling above each pen at the beginning of the trial. This enabled monitoring of the whole pen. Videos were recorded throughout the day, but behaviors were annotated only from videos recorded between 10:00 and 14:00 h of the selected days. This period was selected to minimize disturbances of pig spontaneous behavior (e.g., staff usually entered the nursery early in the morning and in the afternoon, before the end of the day). We focused on days 2, 4, and 6 post-weaning, as these are associated with the digestive disruptions that accompany weaning and the transition from milk to solid feed (4). Thomas et al. (14) have reported that any feeding and drinking behavior differences associated with PWD were observed only during the first week post weaning. The days were thus chosen to detect differences in early changes of pig post-weaning behavior among treatments (4).

#### 2.4.1. Image dataset for method development

Video footage from all six batches was used for this purpose. The collected video footage dataset was sampled into image frames with a sampling rate of 0.05 frame/s. The non-adjacent frame samples were processed and annotated by an expert animal behavioral scientist to establish a representative dataset of that of commercial farms. For instance, the dataset consisted of various scenarios of pigs in close proximity, e.g., when feeding or drinking, in various lighting conditions. A visibility threshold (0.5) was set by the data annotator to perform annotation for any given pig in a frame, i.e., no annotation was allocated to the pig when more than half of its entire body was occluded. The sample frames were also selected to represent all pens used in the study with pigs exhibiting various postures for any given behavior, e.g., a pig standing/drinking and a pig non-standing/drinking. The image dataset also included recordings of RGB (red, green, and blue) and infrared depending on the lighting conditions in a pen at any given time, i.e., poor lighting conditions. The data capturing infrastructure was configured to frequently gauge the lighting condition within each pen and automatically adjust its capturing sensors to adapt to the environment, i.e., switches between RGB and infrared.

To further diversify the image dataset, we configured a set of pre-processing stages by applying random horizontal flipping and arbitrary scaling. We also altered the color of the pixels with selected values of saturation, brightness, and contrast using the HSV color space. The image dataset comprised a total of 8,278 instances (an instance denotes an individual pig label with its bounding box coordinates in a given image) across 810 images. The classes of the dataset were: Standing (2,299 instances across 643 images), non-standing (4,958 instances across 796 images), feeding (622 instances across 365 images) and drinking (399 instances across 370 images). Each pig within an image was manually annotated into one of five behavior/posture categories (see Table 1). By definition, the bounding box specifies the location of a pig within an image. It contains a vector in the format [*x*_*min*_ *y*_*min*_ *x*_*max*_ *y*_*max*_], where *x*_*min*_ and *xy*_*min*_ correspond to the upper left corner of the bounding box while *x*_*max*_ and *y*_*max*_ denote the coordinates of the lower right corner of the bounding box.

**Table 1:** Behavior and posture categories recorded in the dataset and their definitions.

**Behavior**	**Definition**
Standing	At least two hooves are in contact with the floor while the side and the bottom of the pig are not
	The side of the pig is in contact with the floor (lateral lying)
Non-standing-lying	
	The sternum of the pig is in contact with the floor and the limbs are folded under body (sternal lying)
Non-standing-sitting	
	The bottom of the pig and the hooves of the front limbs are in contact with the floor (and the front limbs are extended)
Drinking	The pig snout/snout and forehead touch the bowl drinker
Feeding	The whole head of the pig is in the feed trough

### 2.5. Behavior detection: Training and evaluation procedure

In order to develop a system that can handle the diverse farm conditions (e.g., pigs with different markings and size) and on pens with different settings, e.g., camera orientation and types of recording, we trained the network with many samples of the dataset using pigs exhibiting a diverse range of postures. The training dataset was constructed using sample frames collected at different days/time of the day during trial periods. To evaluate system performance, we used 729 images (including 7,451 instances) for training and the rest 81 for testing (827 instances). Table 2 shows the obtained parameters used to train and evaluate the detection method. These parameters were calibrated with nested cross validation using an independent dataset. The independent dataset was part of the experiment, but contained images that were not used for either training or evaluation.

**Table 2:** Parameter selection used to train and evaluate the detection method; this includes the anchor boxes.

**Parameter**	**Value**
Training step	500, 500
Momentum	0.9
Weight decay	0.5 × 10^−3^
Learning rate (LR)	1 × 10^−3^: with warm-up period of 1 × 10^3^ iteration; this denotes the number of iterations that exponentially increases the learning rate based on the following: $LR=LR\times {\left(\frac{itiration}{warmupperiod}\right)}^{4}$
Max number of epoch	100
Batch size	64
Mini-batch size	8
L2 regularization factor	0.5 × 10^−3^
Confidence threshold	0.5; keep only detections with confidence scores above this value

The selection of parameters (including the learning rate schedule, base network and the additional detection head) was performed using a nested-cross validation using an independent dataset. Mean average precision (mAP) is calculated with an intersection over union (IoU) threshold of 0.5 (or AP50) across all experiments.

### 2.6. Detection method

We adopted several recent well-established models to detect pig behaviors, i.e., You Only Look Once (YOLO) (17–19), centrenet (20), EfficientDet (21), SSD (22), and Faster RCNN (23). The main objective was to configure a system optimized for parallel computations (24–26), with a fast-operating speed and high precision. We utilized several recent versions/configurations of the YOLO architecture (17–19), as they include a bundle of state-of-art modules, i.e., Bag-of-Freebies (denotes methods that control training strategy, e.g., data augmentation (27, 28) and Bag-of-Specials (denotes existing modules, e.g., increasing the size of receptive fields (29) and utilizing attention mechanisms (30). They also use the CSPDarknet53 (31) architecture as a base network which outperformed the CSPResNext50 (31) when tested against well-known datasets such as the MS COCO dataset (32). We have also experimented with a lighter version of YOLOv4, namely, YOLOv4-tiny (17) that uses CSPDarknet53-tiny backbone network instead of CSPDarknet53. The speed of object detection for Yolov4-tiny can reach 371 Frames per second using 1080Ti GPU, which makes it more ubiquitous/portable for real application scenarios, e.g., pig farms.

For evaluation, we utilized the *mAP* (or *AP*^50^) metric. The average precision for a particular class includes both the precision (p) and the recall (r); it denotes the area under the precision-recall curve (Equation 1) across all test image dataset.


(1) $$\begin{array}{l} AveragePrecision\left(AP\right)=\int_{0}^{1}\text{p}\left(\text{r}\right)\text{d}r \end{array}$$

Following the detection stage, we designed lightweight post processing stages to penalize and reduce false positive drinking and feeding instances. For instance, penalizing drinking/feeding detection that takes place away from the drinking/feeding source, i.e., a pig exhibits a similar posture to that of drinking/feeding, however, not in the vicinity of where we expect the corresponding behavior to occur. Here, we utilized the prior knowledge we have about the location of the drinking and feeding sources and designed the penalizing post processing stage accordingly.

### 2.7. Statistical analysis

We analyzed for the following performance and fecal consistency variables: BW, ADFI, FCR, fecal consistency score, and fecal dry matter. For these variables, plots of predicted values against residuals and distribution histograms of residuals were visually inspected to check the homoscedasticity and normality assumptions of models. Data was inspected for outliers by boxplot method. Any outliers were removed from the statistical models (if not specified, this was the reason for sample size reduction). If there was significant effect of any independent variable, *post-hoc* analyzes were used to determine treatment differences (Tukey-Kramer test was applied). The same was done for statistical trends (0.1 < *P* < 0.05). Non-significant effects of explanatory variables were not reported. Data were analyzed in SAS^®^ Studio.

#### 2.7.1. Pig performance

ADFI (kg/d) and BW on days 13 and 40 post-weaning were analyzed by generalized linear mixed models. For ADFI, dietary treatment, the interaction between the treatment and day, day, and average pen pre-weaning BW were included as fixed effects. Pen and batch were included as random effects (Day 13: T1 *n* = 11, T2 *n* = 11, T3 *n* = 11; Day 40: T1 *n* = 12, T2 *n* = 12, and T3 *n* = 12). For BW, dietary treatment, the interaction between the treatment and day, day, pre-weaning BW, and gender were included as fixed effects. Batch and pigs nested within a pen were included as random effects. Two pigs died over the course of the study which resulted in two missing values for BW on day 13 and one missing value for BW on day 40 (Day 13: T1 *n* = 119, T2 *n* = 113, T3 *n* = 119; Day 40: T1 *n* = 118, T2 *n* = 118, and T3 *n* = 119).

FCR (kg/kg) was also analyzed by generalized linear mixed models. Dietary treatment, the interaction between the treatment and day, and day were included as fixed effects. Pen and batch were included as random effects (Day 13: T1 *n* = 11, T2 *n* = 12, T3 *n* = 12; Day 40: T1 *n* = 11, T2 *n* = 10, and T3 *n* = 11).

#### 2.7.2. Fecal consistency and dry matter content

Fecal score was analyzed by generalized linear mixed models (proc GLIMMIX with multinomial distribution and cumulative logit link function in SAS). In the model, dietary treatment, day of measurement and the interaction between the treatment and day, were included as fixed effects. Pen and batch were included as random effects. Any sample reduction was due to lack of fecal samples obtained on the given day (Day 5: T1 *n* = 12, T2 *n* = 11, T3 *n* = 11; Day 12: T1 *n* = 12, T2 *n* = 12, and T3 *n* = 12). Correlations between fecal consistency scores on day 5 and drinking behavior (behavior index, determined below) on either day 4 or 6 were determined by Pearson correlation coefficients. fecal dry matter (%) was analyzed by generalized linear mixed models. In the model, dietary treatment, day and the interaction between the treatment and day were included as fixed effects. Pen and batch were included as random effects. Sample reduction (with one exception) was due to absence feces visible on the floor at the time of sampling (Day 5: T1 *n* = 12, T2 *n* = 8, T3 *n* = 10; Day 12: T1 *n* = 11, T2 *n* = 11, and T3 *n* = 11). Correlations between fecal dry matter on day 5 and drinking behavior on day 4 and 6 were determined by Pearson correlation coefficients.

### 2.8. Automated recorded behaviors

For the behavioral analysis we selected 6 pens per treatment; each pen was selected randomly from each batch to ensure representation. To reduce the effect of the variable frame rate across video footage, we constructed an index as a function of the corresponding cumulative behavior and the total number of frames, see Equation (2). This approach enabled obtaining consistent measures across various data frames across different days and pens of the experimental trial.


(2) $$\begin{array}{l} BI_{i}=\frac{\sum_{k=1}^{N}OBF_{K}}{N} \end{array}$$

In Equation 2, BI_*i*_ refers to a given behavior index, e.g., standing, N is the total number of frames in a video segment, OBF_*k*_ is the occurrence of a given behavior, e.g., standing, at the kth frame. Specifically, a behavior index represents the normalized pen-wise pig with respect to the number of frames per video data.

Behavioral indices for feeding, drinking standing and non-standing per pen were calculated for Days 2, 4 and 6 of the experiment (first week) and analyzed by generalized linear mixed models. In the model, dietary treatment, day of measurement and the interaction between the treatment and day, were included as fixed effects. Correlations between behavioral indices across days were determined by Pearson correlation coefficients.

## 3. Results

### 3.1. Pig performance

Dietary treatment or the interaction between the treatment and day did not have a significant effect on ADFI [treatment: *F*_(2, 30)_ = 0.12, *P* = 0.89; interaction: *F*_(2, 30)_ = 0.25, *P* = 0.78] or BW [treatment: *F*_(2, 345)_ = 0.94, *P* = 0.39; interaction: *F*_(2, 345)_ = 0.96, *P* = 0.39] measured on day 13 and 40. However, there was a significant effect of day and pre-weaning BW on post-weaning ADFI and BW. Pigs consumed more feed [*F*_(1, 30)_ = 2423.71, *P* < 0.0001] and had higher BW [*F*_(1, 345)_ = 10444.50, *P* < 0.0001] on day 40 than on day 13. Pigs which were heavier pre-weaning also consumed more feed [*F*_(1, 30)_ = 9.55, *P* = 0.0043] and were heavier post-weaning [*F*_(1, 345)_ = 206.03, *P* < 0.0001]. There was a tendency for significant effect of dietary treatment on FCR [*F*_(2, 28)_ = 3.08, *P* = 0.06]. Post hoc analyzes revealed that T1 (1.223 kg/kg, *P* = 0.03) and T3 (1.229 kg/kg, *P* = 0.05) pigs had better FCR than T2 pigs (1.287 kg/kg). The treatment x day interaction was not significant [*F*_(2, 28)_ = 0.93, *P* = 0.41], but there was a significant effect of day, as pigs had better FCR on day 13 than on day 40 [*F*_(1, 28)_ = 160.54, *P* < 0.0001]. The results of the performance across the experiment are presented in Table 3.

**Table 3:** The effect of dietary treatment on ADFI (average daily feed intake), BW (body weight), and feed conversion ratio (FCR).

**Parameter**	**Dietary treatment**			**SEM**	***P*-value**

	**T1**	**T2**	**T3**		
ADFI (kg/animal)	0.472	0.476	0.478	0.0297	0.89
BW (kg)	21.1	20.1	21.4	0.353	0.39
FCR (Total feed intake, kg/ BW gain, kg)	1.22	1.29	1.23	0.0221	0.06

Data are presented as LS means with SEM. Weaner pigs received dietary treatment with added ZnO (T1), non -supplemented control (T2) or added antibiotics (T3).

### 3.2. Fecal consistency and dry matter content

There was a significant effect of dietary treatment on fecal consistency score [treatment: *F*_(2, 28)_ = 4.07, *P* = 0.03]. T3 pigs were more likely to have improved (lower) fecal score than T1 and T2 pigs (odds ratios: 0.82 and 2.65, respectively), and T1 pigs were more likely to have improved fecal score than T2 pigs (odds ratio: 3.21). The interaction between dietary treatment and day was not significant [*F*_(1, 28)_ = 0.00, *P* = 0.94]. Dietary treatment did not have a significant effect on fecal DM content [treatment: *F*_(2, 26)_ =1.22, *P* = 0.31], but the interaction between treatment and day tended to be significant [*F*_(2, 26)_ =2.95, *P* = 0.07]. Post hoc analyzes revealed that T2 pigs had lower DM content on day 5 than on day 12 (*P* = 0.04). Moreover, T2 pigs had lower DM content compared to treatment 3 pigs (*P* = 0.02) on day 5 only (232.2, 218.4 and 253.6 for T1-3 respectively); there was no effect of treatment on DM on day12.

### 3.3. Automated detection

**Figure 1:**
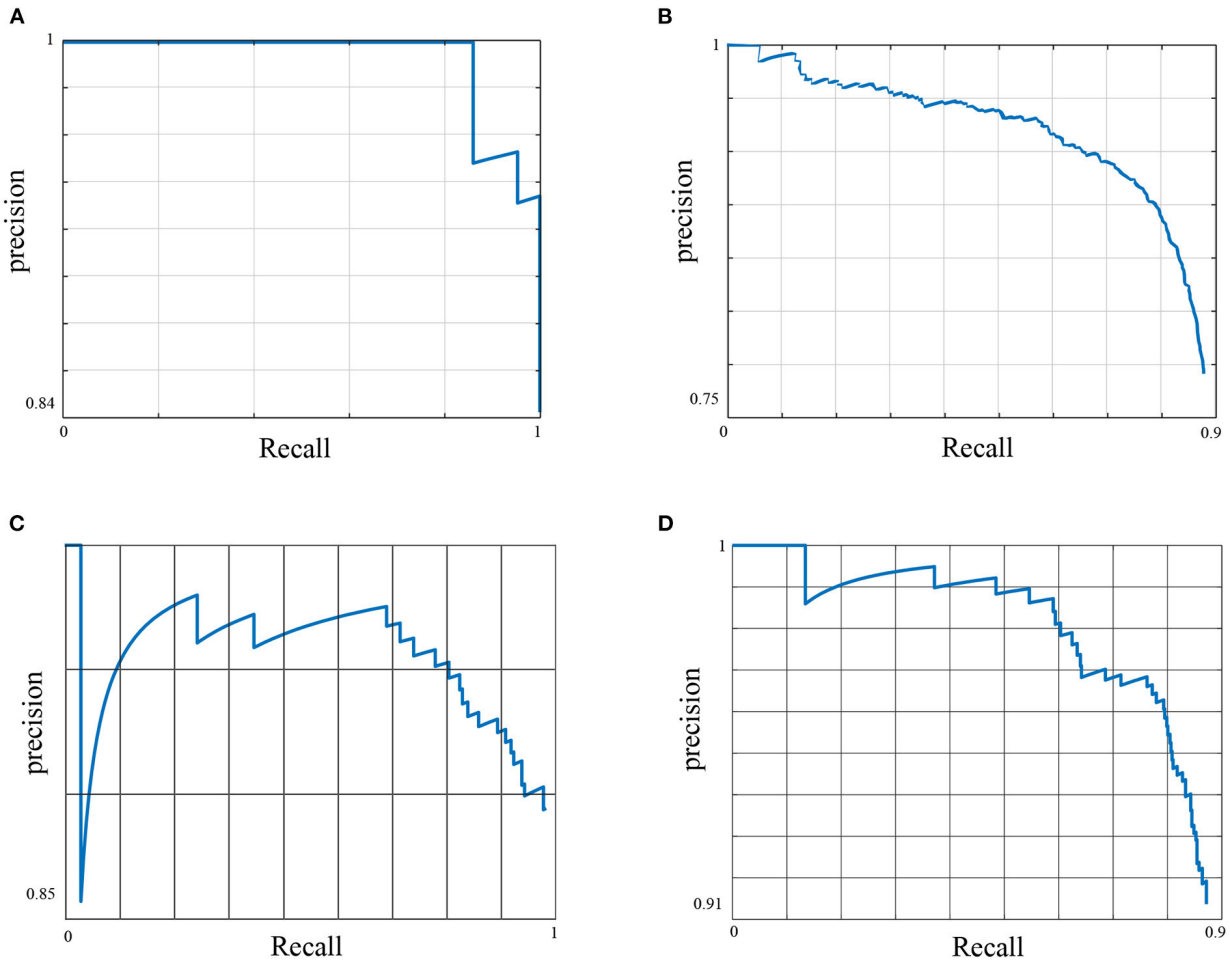
The Precision Recall (PR) curve of the primary model. The figure shows the trade-off between precision and recall for different thresholds for all data classes. A large area under the curve results from achieving high precision and recall, where high precision relates to a low false positive rate, e.g., detecting an instance of the feeding behavior that has not practically occurred, and high recall relates to a low false negative rate, e.g., the method misses an instance of the feeding behavior that has occurred. **(A)** Feeding *AP*^50^ = 0.96, **(B)** Drinking *AP*^50^ = 0.7, **(C)** Standing *AP*^50^ = 0.85, and **(D)** Non standing *AP*^50^ = 0.86.

Table 4 shows the average precision results of the methods utilized in this framework. The YOLOv5 architecture achieved the highest performance across all dataset classes. As a result of that, it was used as a primary platform for the post weaning diarrhea experimental trial quantification of the behaviors and postures of the dataset. The model precision benefited from the post processing (penalizing stage) and its average precision increased by 2% with mean average precision of 0.842. More details about the primary platform performance are provided in Figure 1.

**Table 4:** Parameter selection used to train and evaluate the detection method; this includes the anchor boxes.

**Detection network**	**BackBone**	**Input size**	**AP^50^**
YOLOv4 tiny (18)	CSPDarknet53-tiny (18)	416 × 416	0.633
YOLOv4 (17)	CSPDarknet53 (17)	608 × 608	0.829
YOLOv5 (19)	CSPDarknet53 (19)	640 × 640	0.833
EfficientDet0 (21)	EfficientNet (33)	512 × 512	0.759
centernet (20)	hourglass-104 (34)	512 × 512	0.811
SSD (22)	MobileNetv2 (35)	640 × 640	0.468
Faster RCNN (23)	ResNet-50 (36)	640 × 640	0.652

The selection of parameters (including the learning rate schedule, base network and the additional detection head) was performed using a nested-cross validation using an independent dataset. Mean average precision (*mAP*) is calculated with an intersection over union (IoU) threshold of 0.5 (or AP^50^) across all experiments.

Here, we used the Precision-Recall (PR) curve to inspect primary model performance for each data class. Despite the diverse settings of the feeding sources across different pens of the dataset, the method has shown to detect the feeding robustly with a high average precision of 0.96. The feeding sources were designed to accept an increased number of pigs at any given time, i.e., it did not restrict the number of pigs feeding per feeding trough. The drinking behavior scored the lowest performance across all data classes with an average precision of 0.7. This was predominantly due to the low number of annotated images for this behavior across the entirety of the dataset due to the farm settings, whereby one drinking source was installed per pen, i.e., a maximum of one drinking instance was annotated per data frame. This behavior also does not occur as frequently as some of the other behaviors considered, e.g., standing or lying.

**Figure 2:**
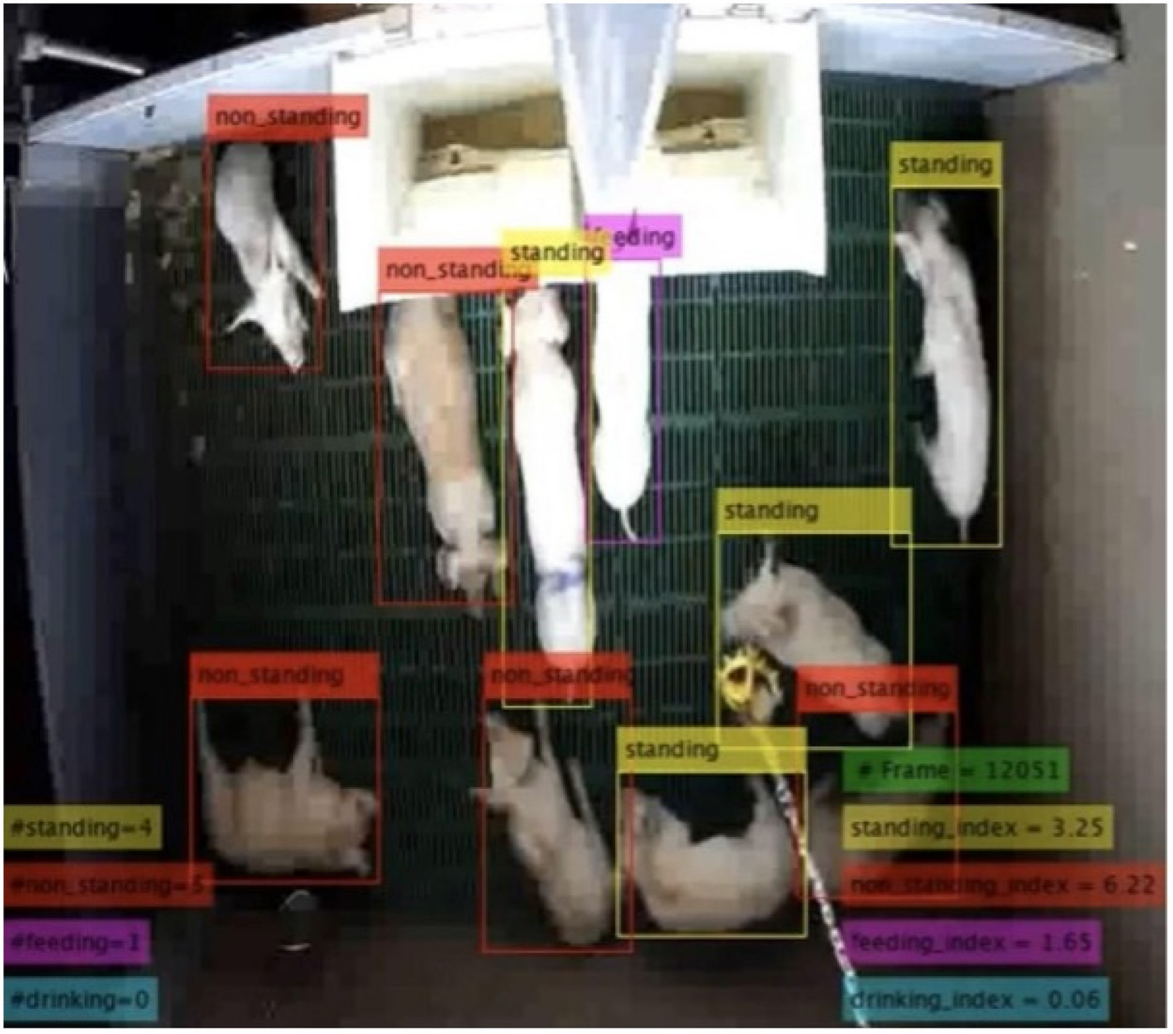
Detection sample for an arbitrary pen of the experimental trial. The method quantifies the behavior of each pig at each time frame. The behavior index is then computed corresponding to the time frame, see Equation (2).

Figure 2 shows a detection sample for a random pen/date/time of the trial life cycle. It shows that the method captures the behavior of each pig within each data frame to accordingly compute an index for any given behavior. The drinking and feeding sources were located at different locations between pens, therefore, the post processing (penalizing) module has reduced the number of false positive detection, mainly for the drinking behavior.

### 3.4. Behavioral indices

**Figure 3:**
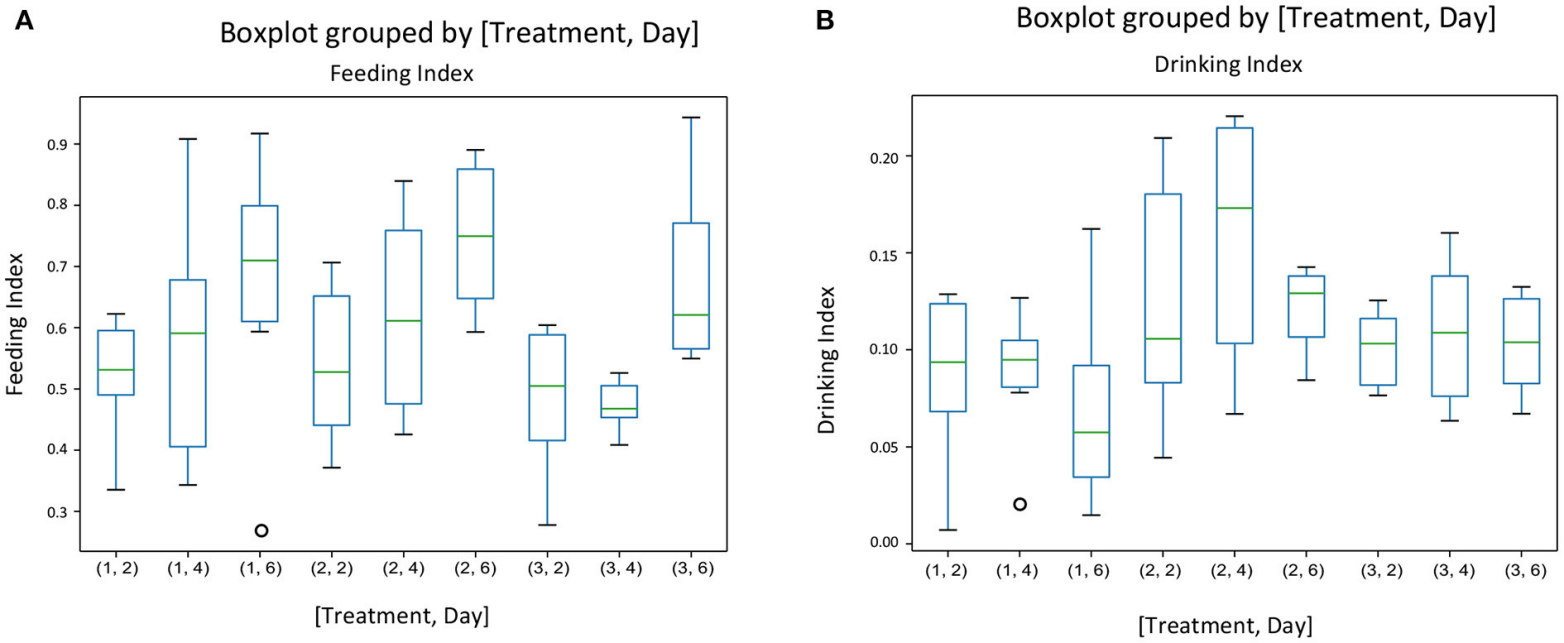
The behavioral indices for feeding **(A)** and drinking behavior **(B)** for each treatment and each experimental day assessed (Days 2, 4, and 6 of the first week of the experiment) in pens of pigs receiving different dietary treatments. Weaner pigs received dietary treatments with added ZnO (T1), non-supplemented control (T2) or added antibiotics (T3). The plots are depicting groups of numerical data through their quartiles. The box extends from the Q1 to Q3 quartile values of the data, with a line at the median (Q2). The whiskers extend from the edges of the box to show the range of the data.

**Figure 4:**
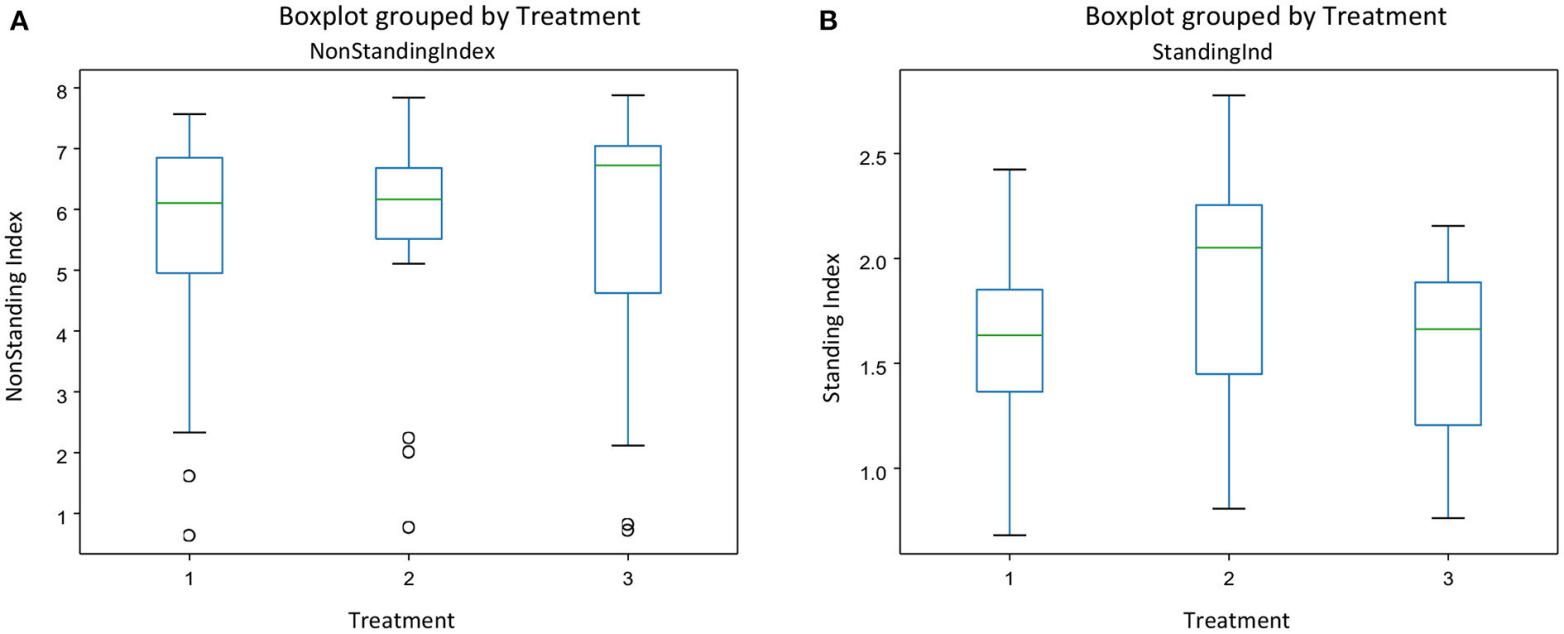
The behavioral indices for Non-standing **(A)** and standing behavior **(B)** for each treatment across experimental days in pens of pigs receiving different dietary treatments. Weaner pigs received dietary treatments with added ZnO (T1), non -supplemented control (T2) or added antibiotics (T3). The plots are depicting groups of numerical data through their quartiles. The box extends from the Q1 to Q3 quartile values of the data, with a line at the median (Q2). The whiskers extend from the edges of the box to show the range of the data.

There was no interaction between treatment and experimental day for any of the behavior indices calculated. Figure 3 gives the mean indices for feeding (Figure 3A) and drinking behavior (Figure 3B) for each treatment and each experimental day The effect of treatment was significant only on the drinking behavior [*F*_(2, 45)_ = [6.05], *p* = 0.004] (Figure 3B). Pigs on the negative control diets (T2) had the highest drinking behavior index, which was significantly different from the index of pigs on the ZnO diets (T1). There were no differences between the three dietary treatments on feeding behavior [*F*_(2, 45)_ = [1.511], *P* = 0.231], but experimental day affected significantly the feeding behavior index [*F*_(2, 45)_ = [7.04], *P* = 0.002], with the values of the index increasing as time progressed (Figure 3A). There were no effects of the experimental day on drinking behavior [*F*_(2, 45)_ = [0.862], *P* = 0.428]. Figure 4 gives the mean indices for standing (Figure 4A) and non-standing behavior (Figure 4B) for each treatment across experimental day, as there were no effects of experimental day on non-standing [*F*_(2, 45)_ = [0.466], *P* = 0.630], and no-standing indices [*F*_(2, 45)_ = [0.091], *P* = 0.912]. Treatment did not significantly affect standing [*F*_(2, 45)_ = [2.955], *P* = 0.06] and non-standing indices [*F*_(2, 45_) = [0.013], *P* = 0.986]. The tendency in the treatment effect on the standing index was due to the higher values for T2 compared to the other two treatments.

### 3.5. Correlation between behavioral index scores

The correlation between fecal consistency and drinking behavior index, and fecal DM and drinking behavior index on day 4 was low (*r* = +0.25 in both cases), but the same correlations on day 6 were strong and positive (*r* = +0.68 and +0.67, respectively). The correlations between the different behavioral indices were relatively low (*r* < +0.30). The only exception was the correlation between the drinking behavior and standing indices, which was moderate (*r* = +0.55).

## 4. Discussion

We used three dietary treatments to manipulate the risk of piglets from enteric disorders in the immediate post-weaning period. It should be emphasized that these three treatments were not meant to relate to practical interventions for the control of digestive disturbances that may arise in the immediate post weaning period, especially in view of the recent ban of prophylactic use of ZnO across the EU (5). They were rather a means to an end. The treatment expected to increase such a risk and penalize pig performance was the one without any in-feed inclusion of ZnO or antibiotic (T2). In practice, withdrawal of in-feed ZnO would be expected to be accompanied by other interventions to reduce the risk arising from the withdrawal, such as reduction in the crude protein content of the starter feed or inclusion of functional fiber (13), which was not the case here.

There was no effect of dietary treatment on the BW and ADFI of the piglets, both in the relatively short (~2 weeks from weaning) and longer term (40 days post weaning). This is consistent with the findings of Li et al. (37) and Slade et al. (38), who did not find any difference in the performance of piglets supplemented with or without prophylactic levels of ZnO, when a similar time scale in the measurement of the variables was considered. The latter ascribed this absence of an effect in the ability of the pigs to compensate in their performance once they overcame the immediate disturbances associated with weaning. More detailed performance measurements suggested that differences between supplemented with ZnO and non-supplemented piglet were evident only up to day 7 post weaning. We observed, however, differences in the FCR of piglets between the three different treatments over the whole experimental period: the lowest FCR was seen in the ZnO supplemented piglets and the highest in the non-supplemented ones. This was also consistent with the findings of Slade et al. (38), who ascribed this effect to the positive effect of ZnO on gut morphological development and its effect on the efficiency of nutrient utilization, which persisted over a period of time.

In addition to performance, we measured fecal DM content and fecal score as proxies of the incidence of diarrhea which might have accompanied the post-weaning transition. Similar to the above, dietary treatment affected fecal DM in the expected direction: non-supplemented piglets (T2) had the lowest fecal DM on day 5, whereas piglets supplemented with ZnO (T1) had the highest. This is consistent with the expected higher incidence of diarrhea in non-supplemented piglets on starter diets similar to the ones used here (1).

Given the above, we were also expecting differences in the behavior of piglets associated with their dietary treatments and the risks they might impose. Such differences may be expected to arise from the development of ‘sickness behavior' (8), which is associated with the development of most infections. Such sickness behavior may be associated with reduced feeding or drinking, reduced activity, and social interaction (39, 40). Alternatively, behavioral modifications may be associated with the pig physiological system affected by the health challenge. For example, a digestive disturbance associated with diarrhea, such as is the case of PWD, may lead to increased drinking and a modification in the pen area used for lying (14). The focus of our paper was to automatically capture these behaviors and investigate their value as a diagnostic tool for the detection of PWD.

We focused on the automated quantification of four behaviors which we considered to be of value in the detection of issues associated with the post-weaning transition in piglets: feeding, drinking, standing and non-standing (please note that standing and non-standing are not necessarily complementary behaviors, as defined in Table 1. This presented us with several challenges for each individual behavior, e.g., drinking behavior, where drinking sources were located at various locations at each pen, depending on the position of the waterpipes. This has been addressed by using a penalizing module which spatially restricts the detection of the drinking behavior to the vicinity of the drinking source in each individual pen. Here, for the first time, we directly detect and quantify the feeding behavior using the detection model and the penalizing module. We do not rely on tracking individual pigs and predict/assume that pigs are feeding when stationed near the feeding troughs or drinking near the drinking source (41). Our approach also eliminates tracking modules and the implications associated with frequent ID switches and directly detect behaviors within the object detection module. This approach can be upscaled smoothly to include more behaviors and postures by using transfer learning, where existing model knowledge can be leveraged by adding new behaviors or similar behaviors from farms with different pen settings to increase model performance. Another advantage associated with this approach is that a false positive or a true negative at one frame does not have implications when processing the next frame as behaviors are collated on a frame-by-frame basis. This solves issues with spatial temporal related approaches whereby a behavior is detected using a set of frames (42). This also includes coping well in scenarios where the data capturing system skips frames due to overheating or when running tasks compete for resources.

The method applied here was able to detect automatically a variety of behaviors with high precision, comparable to the precision detection of behaviors on the dataset where the method was originally developed (10, 11). The only exception was the lower precision for the detection of drinking behavior, which was ascribed to the lower number of annotated images for this behavior. The lack of images was associated with the provision of only one drinking source. This resulted in a maximum of one drinking instance being annotated per data frame, whereas there were several pigs performing the other behaviors per data frame. We assume that the performance of the model in this respect will improve incrementally by adding training images for the less performed behaviors.

The relevant behavior traits were the amount of time spent on the specific behavior over a period of time after adjustment for any variable frame rate. We concentrated on the behavioral changes (behavioral indices) that may happen during the first week post weaning. This focus was justified by the different treatment effects on fecal consistency, which were obvious only during this time frame. PWD is frequently accompanied by inappetence, changes in drinking behavior and changes in posture and locomotion (9, 13). We saw significant changes in two of these behaviors: drinking and standing. The drinking behavior index was increased in the non-supplemented pigs, which is consistent with the suggestion that pigs with PWD may need to drink more water in order to compensate for the water loss associated with diarrhea. For growing pigs, water consumption (drinking flow and frequency) is also a good predictor of diarrhea (43).

The fact that the drinking behavior index was positively correlated with the fecal consistency score and DM content of the feces on day 5 gives support to this suggestion. However, a change in the amount of water drunk is not necessarily associated with PWD or indeed the digestive issues associated with the post-weaning transition (14). This contradiction may be explained by the fact that amount of water consumed and drinking behavior, as defined here (contact between the pig snout and water source), may not necessarily correlate (8).

The increase in the standing behavior index of the non-supplemented piglets, which were assumed to be at higher risk of PWD, may appear in the first instance counter intuitive; we hypothesized that there would be actually a decrease in the standing behavior of piglets with the highest risk of PWD (T2). One of the characteristics of PWD is lethargy which results from inappetence and dehydration. However, Rostagno et al. (44) have reported an increase in standing in pigs infected with an enteric pathogen. It is widely known that pigs preferred to stand whilst defecating, which in pigs with an enteric disorder and diarrhea may contribute toward an increase of the time spent standing. In addition, we identified a moderate correlation between drinking and standing behavior indices. It is therefore possible that, alternatively, the increase in the standing index arose because pigs were actually spending more time drinking, for the reasons discussed above.

Surprisingly, there was no effect of dietary treatment on the feeding behavior index of the pigs. As indicated above, one of the consequences of PWD is a decrease in the daily feed intake during the first week after weaning (13). Indeed, Thomas et al. (14) have suggested that a change in feed intake of pigs suffering from PWD precedes clinical signs of the condition and is exacerbated during the first week post-weaning. Again we would like to emphasize that we did not measure the amount of feed consumed, but the amount of time the pig spent with its head in the feeding trough. This should not necessarily imply that the pig is consuming feed or that the rate of eating is the same between healthy and sick pigs (40). However, we found that the feeding behavior index (and indeed the drinking behavior index) increased with time. This is consistent with the increase in amounts of feed and water consumed as the pigs grow, and should provide further confidence about the reliability of the automated method developed here. The developed automated method for behavior quantification was based on group (pen) averages. Currently, health intervention in pigs occurs at a group level, although it is possible that personalized treatment of individuals whose health is compromised may happen in future (14). Changes in group drinking behavior as an indicator of health issues associated with pig post-weaning transition can be monitored through a more straightforward technology [e.g., water consumption meters (43)], than the one developed here. However, it is recognized that reliance on single technology, such as water flow measurement, has limitations (9). For example, Nathues et al. (45) have suggested that automated cough detection as an indicator of respiratory infection should be combined with seroprevalence measurements and veterinary skills for diagnosis, which of course limits its usability for automation. The method developed here is capable of automatically monitoring several different behaviors in a pen at the same time, and potentially this may be where its value lies. The method is capable of processing up to 5 frames/s without relying on the expensive graphical processing units (GPU). This makes this approach suitable for implementation in low-cost CPU based hardware, e.g., raspberry pi.

In conclusion, we developed a method capable of automatically monitoring several pig behaviors and applied it to detect behavioral changes associated with the post-weaning transition in pigs. We found a number of behavioral changes occurred during this period, and these changes were associated with an increased risk from post-weaning diarrhea. It is therefore possible that the method can be used as the basis for an early warning system, where changes in behavior are used as an alarm to trigger action by the animal keeper to deal with the consequences of enteric disorders in weaned pigs.

## Data availability statement

The codes used for the automated detection of behavior are already publically available in the papers by Alameer et al. (10, 11). The dataset is available upon request by the authors.

## Ethics statement

The work was carried out under the Project License Number PPL2851. All procedures conducted within this study were approved by the Animal Welfare Ethical Review Body and in accordance with the Animals Scientific Act (1986) at the Agri-Food Bioscience Institute (AFBI).

## Author contributions

IK: funding acquisition, project administration and supervision, conceptualization and interpretation of outcomes, and writing—original draft. AA: methodology, software, statistical analysis, and writing—original draft. KB: investigation, statistical analysis and interpretation, and manuscript review and editing. RM: experimental design, resources, and manuscript review and editing. All authors contributed to the article and approved the submitted version.
